# Aligning innovation and security in AI-enabled biotechnology: a framework for designing and funding mutually reinforcing approaches

**DOI:** 10.3389/fmicb.2026.1820739

**Published:** 2026-05-18

**Authors:** Casey Aveggio, Sarah R. Carter, Harshini Mukundan, Elizabeth (Beth) E. Cameron, Steph Guerra

**Affiliations:** 1 RAND Corporation, Arlington, VA, United States; 2 Science Policy Consulting LLC, Arlington, VA, United States; 3 Lawrence Berkeley National Laboratory, Berkeley, CA, United States; 4 Pandemic Center, Brown University School of Public Health, Providence, RI, United States

**Keywords:** AI-enabled biological tools, artificial intelligence, biosecurity governance, biotechnology, dual-use governance

## Abstract

The convergence of artificial intelligence and biotechnology is transforming the life sciences and enabling rapid design, development, and analysis across the research lifecycle. However, this acceleration also heightens the risk for potential biological misuse concerns. Building a sustainable and secure bioeconomy requires moving beyond rhetoric about balancing innovation and security toward practical, operational efforts that do both. This paper proposes a framework for initiatives that embed safety and trustworthiness into the architecture of biological research systems while simultaneously advancing scientific progress. Alongside this framework, we will describe three case studies where investment can yield mutual benefit: (1) secure and tiered data-sharing infrastructures that broaden access to high-quality biological data while maintaining control over sensitive information; (2) provenance and metadata tracking mechanisms for AI enabled biological tools that enhance scientific reproducibility and oversight; and (3) capability benchmarking approaches for AI-enabled biological tools that can enable performance improvements while providing situational awareness about biological risk trajectories. Our framework demonstrates how co-designing innovation and security objectives can transform potential trade-offs into reinforcing outcomes. The paper concludes by outlining policy and funding strategies to enable such mutually reinforcing win-win approaches, positioning responsible AI-enabled biotechnology as both a driver of innovation and a foundation for global biosecurity.

## 1 Introduction

Artificial intelligence (AI) is rapidly transforming the life sciences. Advances in machine learning, large language models (LLMs), and AI-enabled biological tools (BTs)^1^ have had profound impacts on biotechnology, from protein structure prediction and enzyme design to drug discovery and improved diagnostics (Serrano et al., 2024; Chen et al., 2024). Together, these tools are poised to accelerate discovery throughout the research cycle, shortening research timelines that once spanned years into months or even weeks while simultaneously lowering costs. BTs have dramatically improved prediction accuracy and design efficiency, supporting novel therapeutics discovery (Liu Y. et al., 2025), while AI optimization of drug candidate selection, toxicity screening, and clinical trial design has shortened pharmaceutical development timelines (Lee et al., 2025; Dermawan and Alotaiq, 2025). These applications rely on vast amounts of highly accurate and curated biological data, interdisciplinary collaboration, and validated laboratory and digital workflows to enable research efficiency and novel discovery.

However, these same tools and workflows also raise complex questions about safety, security, and governance. AI tools are inherently dual-use, meaning they may lower technical and knowledge barriers to scientific discovery, enable potential biological misuse, and promote the potential for cyberbiosecurity concerns. For example, BTs may enable design of novel pathogens and toxins with unique characteristics capable of evading existing detection systems. Current biosecurity policies are not poised to handle these novel threats, and new approaches will be required to prevent misuse of these tools.

Discussions of the AI and biology intersection are often framed as unavoidable trade-offs between rapid technological progress and security. However, this binary framing is overly simplistic. Innovation and security are not mutually exclusive. The remainder of this paper outlines a framework for identifying and creating opportunities that simultaneously advance scientific progress by removing systemic barriers to innovation while creating new opportunities to strengthen security. Our framework consists of four core principles for the CHALLENGE, STRUCTURE, APPROACH, and COMMUNITY elements of new efforts that have dual benefits. We apply this framework to three case studies demonstrating how this approach can accelerate research while opening new avenues for biosafety, biosecurity, and oversight: (1) secure and tiered data-sharing infrastructures that expand access to curated datasets while protecting high-risk information, (2) data provenance and metadata tracking that enables both research advancement and potential identification of misuse, and (3) continuous evaluation and benchmarking of BTs that informs both tool improvement and security risk assessment. We conclude with a call to action for researchers, funders, and policymakers to intentionally implement this framework into their work, ensuring that AI-enabled biotechnology is both transformative and resilient.

## 2 Background

Since the end of the Cold War, global biosecurity concerns have expanded from state-level biological weapons programs to include terrorist misuse and accidental laboratory releases. Rapid advances in biotechnology, such as DNA synthesis and gene editing, have progressively lowered barriers to biological misuse. Concerns about biological weapons were heightened by events such as the discovery of the massive anthrax production complex in Stepnogorsk, Kazakhstan; the 2001 Amerithrax attacks; and Al Qaeda’s efforts to develop biological weapons in Afghanistan. The advent of AI may increase the number of actors who could design and develop dangerous biological agents and toxins by enhancing access to information, enabling new forms of operational planning, and creating tools for designing novel pathogens (Bengio et al., 2026). Simultaneously, AI is also making it possible to create on-demand vaccines, tests, and treatments for these same biological threats (Attal-Juncqua et al., 2026). Compounding this are cyberbiosecurity concerns, demanding the protection of biological data, systems and materials from digital threats such as hacking and tampering (Mantle et al., 2019; Guttieres et al., 2019).

Biosecurity measures have consistently lagged behind advances in biotechnology, including the emergence of general purpose AI and BTs. Existing national security frameworks are ill-equipped for a world where AI-designed pathogens could become rapidly accessible on-demand. Traditional security approaches—centered on rigidity, information control, and personnel reliability—struggle in an environment where technical knowledge is openly available. In contrast, AI innovation is driven by speed, competition, and limited regulatory oversight. Although some stakeholders have advocated embedding risk mitigation into the earliest stages of research, funders have yet to make commensurate investments in biosafety and biosecurity as integral components of the design–build–test–learn cycle.^2^ Cyberbiosecurity, on the other hand, adds an additional tier of complexity by requiring threat assessment and mitigation at the convergence of biosecurity, cybersecurity and cyber-physical systems.

The solution is not to abandon guardrails, but to ensure that both biodefense and biotechnology evolve to keep pace with AI progress. To effectively prevent, detect, and rapidly respond to biological incidents, it is no longer possible to separate guardrails from innovation. Fit-for-purpose interventions must be designed to strengthen both security and innovation simultaneously.

## 3 A framework for designing and funding mutually reinforcing innovation-security approaches

This paper presents a framework to aid researchers, funders, and policymakers to develop and identify projects that mutually reinforce innovation and security. There are four core principles to this framework that, when implemented effectively, lead to projects that can both accelerate innovation and safeguard national security:

- *CHALLENGE: Problem solving*. Projects must be aimed at solving a challenge in AI-enabled biotechnology that requires urgent action. Solutions-oriented projects will lead to faster support, more allies, and increased research funding.
*Key Question: Does it solve an existing challenge in research or the life sciences community?*
- *STRUCTURE: Integrated security by design*. Projects must embed security features in the research infrastructure, design, or execution to ensure that the project is designed for dual benefit from the start rather than retrofitted at the end. Retrofitting an effort for security purposes can result in delays and may have the unintended effect of reducing buy-in for security-centered innovation approaches.
*Key Question: Does the project embed security features in its design and infrastructure from the outset rather than retrofitting them later?*
- *APPROACH: Innovation-compatible layers.* Projects must consider tiers of access in order to realize the intended innovation and allow for responsible use. The key is to determine which layers could and should be accessible to the widest group in order to accelerate that innovation.
*Key Question: Does the project’s design simultaneously advance innovation while mitigating the highest risk?*
- *COMMUNITY: Diverse stakeholders*. Projects must include engagement of scientists, innovators, national security experts, and policymakers—helping these communities to work together seamlessly and better understand the unique challenges they each face. Such initiatives also will establish a cadre of security and safety focused innovators, creating a reinforcing cycle of professional development for future efforts.
*Key Question: How does the project foster a community of security-focused innovators and innovation-focused security professionals?*

In this paper, we present three project profiles that exemplify this framework and demonstrate its potential for maximizing security-centered innovation and innovation-centered security in practice. These projects are summarized in Table 1 and discussed in greater detail below. Each of these case studies provide similar opportunities to engage a broad range of stakeholders and build a COMMUNITY of biosecurity innovators.

**Table 1:** Summary of mutually reinforcing project profiles.

Project profile	CHALLENGE: Problem solving	STRUCTURE: Integrated security-by-design	APPROACH: Innovation-compatible tiers	COMMUNITY: Diverse stakeholders
Secure and tiered data-sharing infrastructures	A lack of structured and validated data to train and fine tune models for challenging tasks in scientific discovery, research, and development	Systems implement cybersecurity best practices and also incorporate distributed data networks with federated architectures, secure enclaves, and design privacy tools	Tiered access that allows for data with the lowest risk of misuse to be openly available while dual-use or potentially high-risk datasets are provided to legitimate users	Funders and publishers set tiered access norms for how new biology datasets are generated, shared, and accessed by scientific grantees to accelerate future research through open and managed access platforms
Provenance and metadata tracking	Reproducibility in science and need for more effective cross-organization collaboration	Transparency through design mechanisms that make the research process more rigorous and traceable	Most burdensome metadata or provenance tracking only implemented on highest risk tools and workflows	Tool developers, scientists, and biosecurity experts work together to set metadata standards that can be useful to enable scientific progress and potential investigations of misuse
Continuous evaluation and benchmarking	Lack of validated information and data needed to improve and utilize BTs to maximum extent for beneficial research	Built-in mechanisms to assess and share information about the capabilities and risks of BTs	Granularity of evaluation data is stratified by potential risk and impact of misuse, and the users that can benefit from data access and use	Model developers, AI evaluators, biotech and pharma companies work together to develop benchmarks that are relevant to both capabilities and risks. Empirical data is shared to improve model capabilities and inform national security stakeholders

## 4 Case study 1: secure and tiered data-sharing infrastructures

LLMs and BTs need large amounts of highly accurate, curated, and laboratory-validated biological data in order to improve accuracy and reliability of their capabilities and applications (Adalja et al., 2026). The standardization and curation of datasets and associated metadata pose an inherent CHALLENGE in the biological sciences and more effort is needed to generate, annotate, and curate these datasets in order to fully realize the potential benefits of AI-enabled biological sciences. Biology is intrinsically complex and heterogeneous, with variability arising both from biological systems themselves and from experimental processes.

Due to these challenges, there have been significant efforts to generate large biological datasets, yielding major benefits for science. Examples include the U.S. Department of Energy (DOE) user facilities such as the Joint Genome Institute (JGI), which has led to the DOE Knowledgebase, the National Microbiome Data Collaborative, and other applications; CEPI’s Disease X knowledge base in support of the 100 Days Mission (The Disease X Knowledge Base, 2026); and Align, which focuses on AI-ready datasets for biology (Align, 2025). These and other programs have enhanced capabilities to predict and unravel the complexity of biological systems, analyze microbiome data, and accelerate the development of medical countermeasures for pandemic preparedness, among other benefits.

Streamlined data sharing APPROACHES can ensure broader researcher access to high quality annotated datasets, enable larger and more effective AI training in various biological domains, reduce data silos, and offer greater accuracy, efficacy and reproducibility. However, the availability of larger and more curated datasets also increases the potential for deliberate misuse through the open development and/or fine tuning of open weight AI models (Berke et al., 2025). Recent research shows that BTs can generate full viral genomes, including some not found in nature (King et al., 2025). Open-source AI protein design software has been used to generate variants of proteins of concern, which can evade biosecurity screening tools used by nucleic acid synthesis providers (Wittmann et al., 2025). Currently, the limited availability of data and curated datasets with translational relevance to pathogen design and development may limit the probability that an AI tool could generate a threat agent of existential concern. Yet as well-curated, multimodal data become more available—essential for developing pathogen-agnostic surveillance systems and medical countermeasures—the risk of misuse will also grow. Such vulnerabilities must be identified and secured, even as data availability, curation, and integration improve.

Balancing the potential benefits of AI in biotechnology with the risk of misuse not only requires streamlined and standardized frameworks for organizing biological data, but also a carefully designed matrixed STRUCTURE that enables integrated biosecurity and cybersecurity features through tiered access (Carter and Butchello, 2026; Bloomfield et al., 2026). By embedding security into data infrastructure from the outset and stratifying access based on risk, we can accelerate AI-enabled biological innovation while preventing the most dangerous capabilities from becoming widely accessible.

## 5 Case study 2: provenance and metadata tracking for AI-bio tools

The number and diversity of BTs are expanding rapidly, particularly among academic researchers, with many different versions of these tools evolving to fill varied niches in the scientific ecosystem (Webster et al., 2025). In addition to modifying the code, model developers often retrain models with different data, fine-tune them with additional data, and optimize prompts to elicit their desired outputs (Bennett et al., 2026; King et al., 2025). Understanding and reproducing the outputs from a model therefore require accurate information on its provenance, development, and usage. Many of these tools are complex and can infer designs for functional proteins (Hayes et al., 2025; Watson et al., 2023) and whole genomes (King et al., 2025) that are not found in their training data. This lack of standardization represents a key CHALLENGE: how to ensure reproducibility and explainability as BTs proliferate. Further, this novelty can decrease the user’s ability to explain how the model produced the design and increases the need for contextual data to solve this challenge.

As researchers increasingly turn to BTs to process their biological data, tracking metadata generated by these tools will become critical both to support beneficial uses and to ensure biosecurity. Metadata can capture information about a tool and its use, such as provenance, version, timing of use, operations performed, and user inputs. A broadly adopted metadata standard would support scientific priorities including transparency, reproducibility, explainability, and collaborative development, drawing lessons from biological data standards that have enabled critical advances (Mukherjee et al., 2025; Joint Genome Institute, 2026). By capturing metadata alongside the model’s outputs, users can better understand and build on results, while chain-of-custody tracking facilitates scientific discovery and complex workflows across multiple tools, teams, and institutional settings. Embedding transparency mechanisms directly into the tool supports a security-by-design STRUCTURE that enables innovation. Development of a metadata standard requires some investment, and initial implementation of a standard also poses some administrative burden. It will be critical that the adopters of such a standard be supported by funders, life sciences institutions, and the broader innovation and biosecurity communities.

A metadata standard for biological AI tools also strengthens biosecurity by providing more context about an AI-generated biological design. In particular, it can contribute to biosecurity risk evaluations by including information about the users and their inputs into the tool, which may provide insight into their intentions in generating the design. This type of information would be immediately helpful in the context of nucleic acid synthesis screening (Densmore et al., 2025), but can also support biosecurity in other ways. For example, it could enable legitimate researchers to more easily log or register designs, law enforcement to attribute or trace harmful designs, and biosecurity experts to track usage and capabilities of biological AI tools. Metadata requirements could take a stratified APPROACH, allowing for minimal tracking for low-risk applications while more comprehensive metadata capture—including user verification and intent documentation—would apply to tools that have higher risk profiles. Establishing a metadata standard represents a clear example of facilitating legitimate scientific progress while advancing biosecurity priorities. Metadata standards can also include cyber-physical systems, thereby bringing in a core assessment of cyberbiosecurity to this architecture. Indeed, a comprehensive analysis of cyberbiosecurity strategies and schematics shows that our proposed principles are well aligned with academic assessments, largely because agility and adaptability are key drivers in our design process, making it inclusive to new risk categories or data sources (Peccoud et al., 2018).

## 6 Case study 3: capability benchmarking for AI-bio tools

Another key CHALLENGE to progress in AI-enabled biotechnology is that researchers and tool developers lack the ability to reliably benchmark current and future capabilities across the diverse set of BTs. The rapid growth of the tool ecosystem has outpaced our ability to measure each tool’s performance and reliability. This poses challenges for both innovators and security professionals. Without standardized and empirical evaluation methods, developers, investors, and policymakers lack the information needed to improve tools, guide innovation, and anticipate or mitigate emerging risks. Closing this gap can both accelerate discovery and provide grounded evidence to advance our understanding of biosecurity needs.

Similar to how bio-capable general purpose AI models (e.g., LLMs, AI agents) have benefited from a robust benchmarking and evaluation ecosystem (Dev et al., 2025; Mitchener et al., 2025; Liu A. B. et al., 2025), systematic evaluation of BTs will enable researchers to select appropriate tools, drive performance improvements, and establish shared understanding of capabilities and risks. Traditional assessment through wet laboratory validation is resource-intensive and often inaccessible. Scalable and accurate *in silico* evaluation methods—using standardized benchmarks based on lab-validated data (Ikonomova et al., 2025)—offer a transformational path forward. Establishing technically rigorous evaluation methods will ensure that innovation proceeds rapidly on a foundation of trust, transparency, and security. These benchmarks could not only assess risk but also highlight performance strengths, efficiency gains, and areas for technical improvement, creating a feedback mechanism that supports developers in advancing safer, more capable tools.

However, comprehensive benchmarking introduces a dual-use challenge: the same detailed capability information that helps legitimate developers improve their tools could also inform malicious actors about which models are most effective for harmful applications, essentially acting as a ‘blueprint’ for malicious activity. Addressing this tension requires a STRUCTURE for sharing benchmarking results that includes security features such as access controls, usage monitoring, and audit trails. For particularly sensitive information about capabilities of concern, more advanced techniques like confidential computing, secure multi-party computation, or other techniques could also be explored to allow developers to receive feedback without exposing high-risk or sensitive information to the public. It should also follow an APPROACH that implements tiered data release strategies where general performance metrics are openly shared but sensitive capability assessments are restricted to vetted stakeholders who could directly benefit from access such as tool developers, researchers, and security stakeholders. This strategy ensures that innovation proceeds transparently while security-critical information remains appropriately controlled.

## 7 Enabling mutually reinforcing approaches: policy and funding strategies

The path forward for AI-enabled biotechnology is not one of security versus innovation, but their integration to ensure that progress and protection advance together to solve some of science’s greatest challenges. We propose a positive vision for the future of AI-enabled biotechnology where the COMMUNITY—consisting of funders, researchers, and policymakers—prioritize the acceleration of innovation within the development and implementation of its biosecurity projects and proposals rather than as an afterthought.

First, R&D funders must commit resources for innovation-compatible approaches and biosecurity-by-design structures by requiring them as a condition of grant awards, hosting calls for proposals that explicitly prioritize technical means, and treating biosecurity funding as essential infrastructure with reagents, compute, and personnel designated as essential line items rather than separate add-ons. Funders could also establish incentives such as prizes or “moonshot” challenges that equally prioritize innovation and security to establish a race to the top. Top biotechnology donors and investors could publicly announce a dedication to this structure and make long-term commitments to the bioresilient infrastructure needed for AI-enabled biology over the long term, inclusive of biosafety and biosecurity approaches. Second, research institutions could better integrate biosecurity, biosafety, and innovation by providing support, assistance, and incentives for investigators to adopt best practices. Such institutions could provide incentives for researchers to conduct security-conscious research, develop new tools that rapidly advance positive benefits of AI, and evolve their curricula to train emerging innovators in approaches that prioritize more safe and secure designs.

We stand at a pivotal moment. AI-enabled biotechnology is still nascent enough to be built responsibly, with security and safety integrated from the start. This window offers a rare opportunity to establish the norms, infrastructure, and incentives that will define the next generation of biotechnologists and policymakers, while simultaneously positioning the AI-enabled biotechnology field for long-term success. However, if this window is missed, the future could hold the potential for dual use tools and capabilities to proliferate faster than the institutions and policies built to govern them. For example, the public release of a highly capable BT, without safeguards, could be irreversible and could enable a broad range of malign actors to design and develop novel biological agents, including biological weapons. A new norm must recognize our new reality: in a future world where any pathogen that can be dreamed can be designed, defense from large-scale biological catastrophes will depend both on preventing deliberate and accidental release of new agents and accelerating the design of countermeasures for any agent that could come our way. By acting now, we can ensure that the tools and workflows we build to advance the life sciences also safeguard them.

## Data Availability

The original contributions presented in the study are included in the article/supplementary material, further inquiries can be directed to the corresponding author.

## Footnotes

1 AI-enabled biological tools (‘tools’) refer to a wide range of AI models trained on biological data using machine learning techniques. This term encompasses both specialized tools and more general biological foundation models trained on large datasets of biological sequences which can be adapted for a variety of downstream tasks (Webster et al., 2025).

2 For example, the U.S. National Security Commission on Emerging Biotechnology has recommended the establishment of a new hub for biosecurity innovation and oversight within the U.S. Government, housed within the Department of Commerce. Similarly, the International Bio Funders Compact seeks buy-in from life sciences R&D funders to incorporate biosecurity and biosafety considerations into the work they support.
